# Supplementary material for: Insights into herpesvirus assembly from the structure of the pUL7:pUL51 complex
Source: eLife. 2020 May 11;9:e53789. doi: 10.7554/eLife.53789 (PMC7289601; doi:10.7554/eLife.53789)
Supplement: Supplementary file 1. — Contains tables with data collection parameters for the SAXS and X-ray diffraction experiments, list of cross-linked peptides identified by mass spectrometry, and details of the pUL7-pUL51 co-evolution analysis. [file elife-53789-supp1.docx]

**Table S1. SAXS data collection and analysis parameters**. Program version numbers are shown in parentheses.

|  | pUL7:pUL51 | | pUL7:pUL51 (8-142) |
| --- | --- | --- | --- |
|  | 1:2 complex | 2:4 complex | 1:2 complex |
| *Data-collection parameters* |  |  |  |
| Radiation Source | Petra III (DESY, Hamburg, Germany) | | |
| Beamline | EMBL P12 | | |
| Detector | Dectris Pilatus 6M | | |
| Beam geometry (mm) | 0.2 × 0.12 | | |
| X-ray wavelength (nm) | 0.124 | | |
| Sample-to-detector distance (m) | 3.0 | | |
| Temperature (°C) | 20.2 | | |
| Measured *s*-range (nm^−1^) | 0.0225–7.299 | | |
| Exposure time (s) | 0.995 | | |
| Injected protein concentration (mg/mL) | 8 | | 4.5 |
| Shannon-channel limited *s*-range (nm^−1^) | < 5.57 | < 6.70 | < 7.16 |
| *Structural parameters* |  |  |  |
| *I*(0) (a.u.*) [from *p*(*r*)] | 4624 ± 13 | 3136 ± 11 | 3098 ± 5 |
| Real-space *R* g (nm) [from *p*(*r*)] | 4.30 ± 0.03 | 4.79 ± 0.04 | 3.0 ± 0.01 |
| *I*(0) (a.u.*) (from Guinier) | 4538 ± 10 | 3100 ± 7 | 3331 ± 2 |
| *R*_g_ (nm) (from Guinier) | 3.95 ± 0.07 | 4.56 ± 0.28 | 2.96 ± 0.01 |
| *D*_max_ (nm) | 18.2 | 19.7 | 11.5 |
| Porod volume estimate (Vp, nm^3^) | 160 | 340 | 116 |
| *Molecular-mass determination* |  |  |  |
| Molecular mass *M*_r_, kDa [from SAXSMOW] | 95 | 177 | 73 |
| Molecular mass *M*_r_, kDa [from Vc] | 81 | 167 | 66 |
| Molecular mass *M*_r_, kDa [from Bayesian consensus] | 86–96 | 162–195 | 66–73 |
| Expected *M*_r_  from sequence, kDa | 84.37 | 168.7 | 63.11 |
| *Software employed* |  |  |  |
| Primary data reduction | *CHROMIXS* | | |
| Data processing | *PrimusQT/GNOM(5.0)* | | |
| *Ab initio* analysis | *DAMMIN(5.3)/GASBOR(2.3i)* | | |
| Spatial averaging and resolution estimates | *DAMAVER(5.0)* | | |
| Computation of model intensities | *CRYSOL(2.8.3)* | | |
| Pseudo-atomic modelling | *CORAL(1.1)* | | |
| *Small Angle Scattering Biological Data Bank* |  | | |
| SASBDB accession codes | SASDG57 | SASDG47 | SASDG37 |

*arbitrary units

**Table S2. Crystallographic data collection and refinement statistics**. Statistics for the highest-resolution shell are shown in parentheses.

|  | Native | Mercury(II) acetate derivative | | | |
| --- | --- | --- | --- | --- | --- |
|  |  | Remote (High E) | Peak | Inflection | Remote (Low E) |
| *Data collection* |  |  |  |  |  |
| Wavelength (Å) | 0.97625 | 0.99702 | 1.00728 | 1.00809 | 1.01627 |
| Space group | *P* 2_1_ | *P* 4 2_1_ 2 | | | |
| Cell dimensions |  | | | | |
| *a, b, c* (Å) | 79.51, 106.3, 106.0 | 106.5, 106.5, 79.0 | 106.5, 106.5, 79.2 | 106.5, 106.5, 79.3 | 106.5, 106.5, 79.4 |
| *α, β, γ* (°) | 90, 92.0, 90 | 90, 90, 90 | 90, 90, 90 | 90, 90, 90 | 90, 90, 90 |
| Resolution (Å) | 105.9–1.8 (1.86–1.83) | 53.2–2.8 (2.96–2.80) | 53.2–2.9  (3.02–2.87) | 53.3–2.9  (3.09–2.91) | 53.3–3.0  (3.16–2.98) |
| Unique reflections | 154,984 (7654) | 11,618 (1627) | 10,931 (1561) | 10,498 (1643) | 9789 (1515) |
| Completeness (%) | 100 (99.9) | 99.7 (97.9) | 99.9 (99.5) | 99.9 (99.7) | 99.7 (98.5) |
| Anomalous | – | 99.7 (97.9) | 99.9 (99.5) | 99.9 (99.7) | 99.7 (98.5) |
| Multiplicity | 6.5 (6.5) | 47.6 (36) | 24.9 (25.6) | 24.8 (24.9) | 24.7 (24.8) |
| Anomalous | – | 25.9 (19.1) | 13.5 (13.5) | 13.5 (13.2) | 13.5 (13.2) |
| *R_merge_* | 0.120 (3.197) | 0.261 (2.664) | 0.231 (2.506) | 0.235 (2.348) | 0.232 (2.158) |
| *R_pim_* | 0.051 (1.367) | 0.038 (0.435) | 0.047 (0.502) | 0.048 (0.476) | 0.047 (0.439) |
| CC_1/2_ | 0.996 (0.321) | 0.999 (0.804) | 0.999 (0.783) | 0.999 (0.792) | 0.999 (0.809) |
| CC_anom_ | – | 0.830 (0.059) | 0.741 (0.000) | 0.664 (0.014) | 0.224 (0.000) |
| Mean I/σ(I) | 6.8 (0.5) | 14.3 (1.7) | 12.1 (1.6) | 12.4 (1.7) | 12.4 (1.8) |
| Wilson B (A^2^) | 35.2 |  |  |  |  |
| *Refinement* |  |  |  |  |  |
| Resolution (Å) | 26.6–1.8 (1.84–1.83) |  |  |  |  |
| Reflections |  |  |  |  |  |
| Working set | 154,885 (2892) |  |  |  |  |
| Test set | 8093 (206) |  |  |  |  |
| *R*_work_ | 0.194 (0.240) |  |  |  |  |
| *R*_free_ | 0.220 (0.244) |  |  |  |  |
| No. of atoms |  |  |  |  |  |
| Protein | 11692 |  |  |  |  |
| Solvent | 717 |  |  |  |  |
| Other* | 28 |  |  |  |  |
| Root mean square deviation |  |  |  |  |  |
| Bond lengths (Å) | 0.009 |  |  |  |  |
| Bond angles (°) | 0.90 |  |  |  |  |
| Ramachandran favoured (%) | 98.7 |  |  |  |  |
| Ramachandran outliers (%) | 0.0 |  |  |  |  |
| Poor rotamers (%) | 0.39 |  |  |  |  |
| Mean B value (A^2^) | 45.2 |  |  |  |  |

*Glycerol molecules and chloride ions

**Table S3. pUL7:pUL51(8–142) cross-links identified by mass spectrometry.** Two protein bands from SDS-PAGE analysis, with molecular masses corresponding to 1:1 and 1:2 pUL7:pUL51(8–142), were analyzed.

| Reagent | Band | Protein | Residue | Sequence | Protein | Residue | Sequence |
| --- | --- | --- | --- | --- | --- | --- | --- |
| DSBU | 1:1 | pUL7 | 17 | AAATADDEGSA-  ATIL[**K**]QAIAGDR | pUL51 | 107 | HHPGLEAPTIDG-  AVAAHQD[**K**]MR |
| DSBU | 1:1 | pUL51 | 67 | RLV[**K**]AR | pUL51 | 67 | LV[**K**]AR |
| DSBU | 1:1 | pUL7 | 285 | APLVYWWLSETP[**K**]R | pUL51 | 67 | LV[**K**]AR |
| DSBU | 1:2 | pUL51 | 67 | RLV[**K**]AR | pUL51 | 67 | LV[**K**]AR |
| DSSO | 1:1 | pUL7 | 91 | FVLDGSPEDAYVTSEDYF[**K**]R | pUL51 | 67 | LV[**K**]AR |
| DSSO | 1:1 | pUL7 | 17 | AAATADDEGSA-  ATIL[**K**]QAIAGDR | pUL51 | 107 | HHPGLEAPTIDG-  AVAAHQD[**K**]MRR |
| DSSO | 1:1 | pUL51 | 107 | HHPGLEAPTIDGAVAAHQD[**K**]MR | pUL51 | 67 | RLV[**K**]AR |
| DSSO | 1:1 | pUL7 | 163 | SHATPSTFA[**K**]VLAWLGVAGR | pUL51 | 67 | LV[**K**]AR |
| DSSO | 1:1 | pUL51 | 131 | LADTCMATILQMYMSV-  GAAD[**K**]SADVLVSQAIR | pUL51 | 67 | RLV[**K**]AR |
| DSSO | 1:1 | pUL7 | 17 | AAATADDEGSA-  ATIL[**K**]QAIAGDR | pUL51 | 67 | LV[**K**]AR |

**Table S4.** **Co-evolution of the pUL7-pUL51 interaction interface across *Alphaherpesvirinae*.** *z* is the sum of correlation values for interacting residue pairs and *p* is the probability that this value would be expected by chance. The selection of sequences for each data set is described in *Materials and Methods*.

|  |  |  | No. of interacting residues | | |  |  |
| --- | --- | --- | --- | --- | --- | --- | --- |
| Data set | No. strains, *N* | No. interactions, *I* | | pUL51 | pUL7 | *z* | *p* |
| 1 | 199 | 35 | 21 | | 19 | 4301.24 | 0.061 |
| 2 | 197 | 54 | 27 | | 29 | 7319.44 | 0.068 |
| 3 | 197 | 52 | 26 | | 28 | 6969.40 | 0.065 |
| 4 | 197 | 38 | 22 | | 21 | 4559.47 | 0.062 |
